# Scientific integrity: critical issues in environmental health research

**DOI:** 10.1186/1476-069X-7-S1-S9

**Published:** 2008-06-05

**Authors:** Domenico Franco Merlo, Kirsi Vahakangas, Lisbeth E Knudsen

**Affiliations:** 1 Epidemiology and Biostatistics, Istituto Nazionale per la Ricerca sul Cancro, Genoa, Italy; 2 Department of Pharmacology and Toxicology, University of Kuopio, Kuopio, Finland; 3 Environmental Health Institute of Public Health, University of Copenhagen, Denmark

## Abstract

Environmental health research is a relatively new scientific area with much interdisciplinary collaboration. Regardless of which human population is included in field studies (e.g., general population, working population, children, elderly, vulnerable sub-groups, etc.) their conduct must guarantee well acknowledged ethical principles. These principles, along with codes of conduct, are aimed at protecting study participants from research-related undesired effects and guarantee research integrity. A central role is attributed to the need for informing potential participants (i.e., recruited subjects who may be enrolled in a study), obtaining their written informed consent to participate, and making them aware of their right to refuse to participate at any time and for any reason. Data protection is also required and communication of study findings must respect participant's willingness to know or not know. This is specifically relevant for studies including biological markers and/or storing biological samples that might be analysed years later to tackle research objectives that were specified and communicated to participants at the time of recruitment or that may be formulated after consent was obtained.

Integrity is central to environmental health research searching for causal relations. It requires open communication and trust and any violation (i.e., research misconduct, including fabrication or falsification of data, plagiarism, conflicting interests, etc.) may endanger the societal trust in the research community as well as jeopardize participation rates in field projects.

## Note

*"...a moltitudine de' veri concorre all'investigazione, accrescimento e stabilimento delle discipline, e non alla diminuzione o destruzione*,...'

''...*the multitude of known truths stimulates the investigation, growth, and establishment of the disciplines, not their diminution or destruction*,...''

Galileo Galilei

Lettera a Cristina di Lorena, Granduchessa Madre di Toscana. Opere, Edizione Nazionale a cura di Antonio Favaro, Giunti-Barbera, Firenze 1968, vol. 5, pp. 309-348.

## Environmental health research

### Why is scientific integrity important in environmental health research

The field of environmental health science [1], is an interdisciplinary field, a discipline requiring cooperation between scientists trained in different fields, from basic science (e.g., biology, physics) to toxicology, industrial hygiene, occupational health, epidemiology, analytical and organic chemistry, statistics, and molecular biology. Environmental health interacts with social, political and economical forces and may have a high social and economic impact, which in turn leads to pressure on the scientists working in the area [1]. Because of this it is important to actively foster objective, independent, and rigorous scientific work to enhance our understanding of environmental health. As for clinical research, environmental science requires open communication and trust and any violation against scientific integrity may endanger the societal trust in the research community and eventually jeopardize participation rates in field projects. It may also affect seriously the public-science trustful relationship and preclude lay people from participation in research so that studies may not be any more representative of the target population.

### Ethics within and between research groups

Good scientific practice in large epidemiological health studies is supported by good communication between and within the research groups with everybody carrying the responsibility also of the original data (at least by knowing how it was collected, where it is stored, and how it has been used for conclusions). This is not as self-evident as one might assume judging by the recent examples of revealed fraud cases [2,3]. Especially a case of Jon Sudbo [4,5], who's publications were proven to be invalid because of the fabrication and manipulation of data, revealed the possibility to misuse of colleagues in such cases through international collaboration. In addition to the real harm to colleagues, the results of data falsification are manifold from distracting the scientific community from real research priority, diverting public agency funds from useful to useless research programmes, affecting regulatory standards, undermining trust on research, and, at the personal level, meaning the end of a career along with possible application of penalties [6,7]. Data fabrication and falsification may appear also in documents submitted to funding agencies to obtain research grants in addition to papers in scientific journals or presented at public meeting or used as scientific evidence in patent application [8]. In all these cases greed for money and fame has overruled scientific integrity and damaged trust on collaborators which is essential in scientific community to carry out large studies. In epidemiological studies the effect of a poor design cannot be corrected through statistical analysis. Therefore, cooperation with statisticians is important already at the planning stage with identification of the study design and the preparation of the written study protocol. A clear differentiation between aims and hypotheses formulated before data collection, and analysis and interpretation of the study findings and their publication thereafter, central to science, should be agreed within the whole group carrying out the research. Stating the biological relationships under investigation, the main and secondary research hypotheses, identifying the target population and the study samples, and the statistical methods to be used for formal hypothesis testing is essential. Collaboration already at this stage also helps to prevent potential selective data censoring and torturing [9]. The larger the project is the more contributors there are leading to higher number of authors especially in multidisciplinary studies. Currently, a small minority of research papers are written by one author only [10]. At the publication phase the question of authorship may become a controversy within a multidisciplinary research project [10]. The practices vary between disciplines and no general guidelines exist except that each author must have participated in each stage of research enough to deserve authorship and be responsible for the content of the paper [10,11]. To avoid difficulties at the publication stage, it is recommendable to plan for publication policy already at the stage of planning [11,12]. Erlen and coworkers [11] present an example of such guideline of authorship and stress the importance of including resolution of conflicts to be included.

## Scientific integrity

### What is scientific integrity?

Integrity has been defined as "a steadfast adherence to a strict moral or ethical code" [13]. According to the Institute of Medicine (IOM) for the individual scientist, *"integrity embodies above all a commitment to intellectual honesty and personal responsibility for one's actions and to a range of practices that characterize responsible research conduct. It is an aspect of moral character and experience*." Within a research institution, integrity is *"a commitment to creating an environment that promotes responsible conduct" *[14]. Clearly, both the individual investigators and the research environment are expected to act with integrity to guarantee scientific standards of excellence, trustworthy research findings, and to preserve public confidence in biomedical sciences.

The integrity of science is intrinsic to the scientific method and consists of a set of principles and tools aimed at ensuring unbiased testing of scientific theories (e.g., study hypothesis). The foundation of the scientific methods is the "hypotheses statement and testing": when possible explanations of a phenomenon are proposed, experimental studies are required to test these hypotheses. It follows that other scientists in independent research settings should be able to replicate the findings of a given study within the variability allowed by random or systematic measurement errors. Indeed, repeating the experiments is a commonly accepted research practice. Failure to replicate specific findings may question or disprove the hypothesis. In this process honesty and trust on fellow scientists is taken as self-evident. Fraudulent behavior, when found, always leads to the end of career. Unfortunately, it also affects the trust of public for science which is a prerequisite for participation, and is required to finance research and to make use of research findings in society [15].

Several types of inappropriate behavior have been identified as capable of undermining research integrity [16], most of which fall into the following areas: unethical treatment of research subjects, fabrication and/or falsification of data, plagiarism, and failure to disclose conflict of interest. These practices apply to all typical phases of biomedical research: proposing the study hypothesis and the methods for scientific approval, competing for funding, conducting the research and the formal statistical analyses which have been declared in the research protocol, interpreting and reporting the findings. They also apply to the many roles undertaken by most scientists who may act contemporarily as researchers, research reviewers for funding agencies and or scientific journal, members of institutional review boards, research ethics committees, governmental bodies, national or international committees, consultants for chemical or drug companies and consultancy firms, or present expert testimonies at trials. Their participation in the field of biomedical research results in a complex net of connections and relationships that extend well beyond the field of science with their aims (i.e., the advancement of knowledge, promotion of health and well-being), with public health, regulatory and legal implications.

### Ethical treatment of study participants and their biological specimens

Biomedical research has to apply the well acknowledged ethical principles concerning people who participate in biomedical scientific studies. These principles were identified some 50 years ago [17] and have been developed further [18,19] to meet the needs of the fast growing, evolving biomedical field. The principles are aimed at protecting study participants from research-related adverse-effects and guarantee research integrity. Within this ethical framework, scientists have an obligation to study subjects as well as their biological specimens [20] and must conform to the ethics of the medical profession to satisfy moral, ethical and legal concepts. In order to meet ethical standards, environmental health research must be socially and scientifically valuable. Only scientifically valid research can provide, what is expected from environmental health research as a branch of biomedicine: sound scientific evidence for primary prevention.

For epidemiological environmental health research the establishment and use of large biobanks would be very useful and various efforts in that direction over the past 10 years have been initiated [21]. With such initiatives, the ethical aspects, especially those in connection with the ethical treatment of donators and their samples, have emerged. The idea of a biobank is to systematically collect and store identifiable biological material or samples from which protein, DNA and other elements can be isolated, and then connect biological data with individual data about health and life-style. This is not possible without an extensive use of information technology which may endanger privacy [22]. The discussion of almost all other ethical aspects, e.g. management of biobanks, informed consent issues and financial aspects are linked to the privacy issue [23].

### The principle of autonomy and the informed consent

Important aspects of autonomy are informing potential participants (i.e., recruited subjects who may be enrolled in a study), obtaining their written truly informed consent to participate, and making them aware of their right to refuse to participate at any time and for any reason. However, this is not sufficient for autonomy. Study participants should also have the right to privacy both as to data protection and whether to know their own results if any clinical or experimental assay is performed as part of the research protocol. This is especially relevant for studies including vulnerable subjects (e.g., children, impaired people), measuring biological markers, and/or storing identifiable biological specimens (biobanking). Samples from biobanks may be analyzed years later to tackle research objectives that were specified and communicated to participants at the time of recruitment or that may be formulated after consent was obtained. Rules securing recovery of samples, but also preventing potential misuse need to be set up. The Data Protection Directive [24] states that safe handling of keys for sample codes must be assured by minimising the number of persons with access to this information. In the case samples are forwarded to other institutions it would be advisable to keep the code at the sample collector and/or double code the samples. As stated in the pharmacogenetics terminology paper developed by the European Medicines Agency, long term storage of identifiable samples enabling follow-up studies is necessary for full term development of safety assessment of medicines [25].

Thus the Agency recommends broader scopes of the informed consent and keeping identification of study persons but data protection by double coding. Obtaining a truly informed consent is quite complicated when vulnerable subjects and people with diminished capacity of self-determination (i.e., limited autonomy) are recruited. This is especially the case with children whose ability to consent is age dependent, being related to physical, mental, and psychological development [26]. Indeed, children may not be fully capable of understanding complex biomedical research issues. It follows that no true consent can be obtained from children during their gestational life and during their infanthood. However, children from the age of 3–4 can already be explained using proper language, what will be going on, and assent may be obtained. Parental advice with their informed permission along with the child's assent is the right of children and fulfils the autonomy [26,27]. Such a principle includes the right of the child to opt out when legally adult.

This is most relevant for the EU-biomonitoring program where samples from different exposure groups may provide useful information about past exposures to hitherto unknown exposures to new compounds. Some argue that such new studies need not only a renewed ethics approval but also a new informed consent from the participant [28]. For the biomonitoring programs a system ensuring optimal use of samples is needed – it can be argued that it is not ethically justifiable to collect new samples if suitable samples exist in a biobank. How this can be accomplished without violating the autonomy of the study participants to decide current and future use of their samples has not been resolved. As a pragmatic solution a broad informed consent (prospective consent) for the core study and future uses approved by an ethics committee has been proposed [29,30].

Studies on children are scientifically justified by a possibly increased susceptibility of children (and fetuses) to environmental genotoxic and immunotoxic agents [31]. By comparing groups with different exposures to common environmental pollutants (e.g., mainstream and environmental tobacco smoke, food, ground and water contaminants) and with different susceptibilities to such pollutants, the knowledge of the risk posed by environmental exposures is expected to be enhanced. Consequently, age-specific regulatory actions may be established based on the identification of safety levels for children.

## Research misconduct

### What constitutes research misconduct

Cases of misconduct in medical sciences have been documented and concern both clinical and environmental research [32-38]. Although misconduct is a term commonly used to describe deviant scientific behavior, it is difficult to define, and there has been pressure to use a definition which is the "lowest common denominator" of the term: fabrication, falsification and plagiarism, but to leave out e.g. misbehavior and waste [39,40]. In this context it is important to realize that fraud is never the first step fraudulent scientists do, as Check (2002) reminds [41]. Getting away with small unethical deeds may lead to bigger issues. At the other end is a strict regulation of scientific community. Richman and Richman (2007) propose an approach for preventive measures simulating those in commercial companies [42]. However, this could lead to structures that paralyze innovative research (all methods, data and processes audited by external bodies) but at the end fail to rule out scientific misconduct, because no one person could be responsible as an "accountable scientist" for research integrity, as they propose.

In 1999, a consensus statement of the Council of Science Editors (CSE) provided a broad definition of research misconduct: *behavior by a researcher, intentional or not, that falls short of good ethical and scientific standard *[43]. The result of such a behavior can affect research to the extent that the findings reported by a researcher no longer reflect the observed truth [44], data publication is intentionally deferred [45], or suppressed [33,46]. CSE refined the definition of misconduct in 2002 by specifying that two concepts are central to scientific misconduct: deceit and negligence [20] and excluding honest errors from the definition. Such a position is based on the Medical Research Council's policies and procedures for inquiring into allegations of scientific misconduct [47] and the guidelines of the largest biomedical charity in the UK, the Wellcome Trust, which does not consider honest error or differences in the design, conduct and interpretation or judgment in evaluating research methods or findings a misconduct [48]. In other words, only if poor-quality methods are used with the intention to deceive or without regard to patients' safety, researchers' behavior can be considered not conforming to prevailing standards. All the other cases of poor-quality research are simply considered as poor science. Does this imply that poor-quality science can be funded and published? Actually an important aspect that research ethics committees have scrutinized is the very quality of the research with the understanding that it would be waste of public money to carry out poor research.

It is clear that there is an urgent need for a clear definition of misconduct considering that individual researchers, institutions, funding agencies, journals, scientific societies, governments, the society components such as health and environmental priorities, and, last but not least, the public trust in science are all dependent on scientific integrity[14,49]. It seems clear also that any operational definition identifying faulty behaviors, including a preliminary taxonomy already available [50] is subjected inevitably to continuous reviews according to the development of science as well as ethics of science [51,52]. The variability and the lack of consistency of the definitions of behaviors constituting misconduct [40,50,53] are of no help to researchers worldwide. It is within this complex scenario that the First World Conference on Research Integrity organized by the European Science Foundation (ESF) and the US Department of Health and Human Services, Office of Research Integrity (ORI) was held in Lisbon, Portugal on 16 to 19 September 2007 [54]. The ESF-ORI Conference has focused attention on the lack of agreement on key elements of research behavior and wide variation implementing research policies reported between countries and organizations "*in order to retain public confidence and to establish a clear best practice frameworks at an international level" *[2,54].

### Fabrication and or falsification of data

Data fabrication means making up data or other relevant information at any stage of the typical scientific process spanning from research development and application for funding, up to the submission of findings for publication [20]. Falsification indicates alterations or intended misinterpretation of the true evidence of experimental or observational studies. Falsification is in a continuum state with the process of data selection, the latter implying declared and defendable criteria based on the aims of a given research and the statistical methods identified to test main and secondary hypothesis as stated in the written research protocol. Falsification is perpetrated when data selection, is carried against or without any scientific or statistical justification. Data fabrication and falsification are punishable, when there is evidence that false information are incorporated into official document that are submitted to funding agencies to obtain research grants, scientific journals to disseminate knowledge, when they are presented at public meeting or used as scientific evidence in patent application. The results of data falsification may be manifold from distracting the scientific community from real research priority, diverting public agency funds from useful to useless research programmes, affecting regulatory standards, undermining trust on research, and, at the personal level, meaning the end of a career along with possible application of penalties [6-8,55].

### Plagiarism

Intentionally using other's people thoughts, ideas, or words is considered plagiarism. It is a violation of the ethical principle of failing to reveal and crediting an existing source (a practice that dates back to Euclid, 300 BC) by proposing as one's own idea when it truly belongs to someone else. It is a matter of false attribution. Self plagiarism is also considered ethically unacceptable: it occurs when one uses he's own previously published work (or parts of it) without citing any source. Self plagiarism rather than being strictly an issue of false attribution is generally a way of increasing someone's scientific production by attempting to repeatedly publish copies of one's own research findings or papers in different scientific journals. Although scientific journals' publication policy requires that a paper can be submitted for publication to a single journal [56], redundant publication appears to be a serious problem in biomedical sciences with the frequency of duplicate publication ranging between little less than 10% and 14% [57,58]. Plagiarism in biomedical research may be reduced by collaboration and multiple authorship and prevented by implementing the practice of mentoring within research teams and research institution [59].

### Failure to disclose conflict of interest (CoI)

A conflict of interest (CoI) arises when an individual's interests may compromise communications to research subjects, research reports (dissemination) [60], and in situations when a researcher has competing personal obligations or financial interests that would interfere with a researcher objectivity or when a reviewer has an interest in a grant application that is likely to result in a biased review. Therefore, CoI can occur in any situation in which there is increased potential for the violation of the accepted norms governing responsible research conduct and the professional obligations and commitments to universities or research institutions. Friedman [61] attributes to CoI "an erosive effect on trust in science", capable of undermining the society trustful attitude toward science (i.e., scientists and research findings). The importance of knowing conflict of interest information (transparency) is considered relevant by the majority of potential research participants, with 64% to 87% of them indicating that financial CoI should be disclosed as part of informed consent [62,63]. Indeed, CoI has been shown to significantly interfere with researchers' objectivity in environmental health research [64]. Examination of 106 review articles on passive smoking showed that the author affiliation with the tobacco industry was the only factor associated with the conclusion against the evidence that exposure to passive smoking is associated with health risks [64]. The practice of funding academic scientists through *ad hoc *created industry-supported research projects lacking independency is well known [65]. The result of such misconduct is a seriously biased piece of scientific evidence undermining public trust in science and precluding or postponing effective preventive actions [66]. These twofold implications require that full transparency and accountability is enforced within research institutions and research funding agencies to ensure that potential conflict of interest do not arise or are effectively managed. The role played by each party in the generation, reporting and interpretation of research findings must be clearly stated to enable the scientific community as well as the general public understanding the relationship between individual investigators, academic institutions, private and public funding agencies, and industry.

Recently a perception study of mothers donating placentas for experimental research showed general trust in research, with face-to-face interaction, written information material and informed consent forms playing an important role in creating trusting relationships in medical research [63].

In most European regulations regarding research ethics committees the applicant is asked to include information about potential conflicts of interests. A CoI should be declared also during the evaluation of research projects submitted for funding to the European Commission Research Framework Programmes. Since an unbiased evaluation of research projects is required, the appointed independent experts have to sign a declaration certifying that they have no conflict of interest at the time of appointment and that they will inform the Commission if any CoI should arise [67].

The U.S. National Institutes of Health, Office of Extramural Research has established regulations to ensure that Government employees, scientific reviewers, or others having the ability to influence funding decisions have no personal interest in the outcome of a review process of any NIH-supported investigation. Reviewers are required to certify that they have disclosed all conflicts of interest they may have with the research applications or contract proposals [68,69].

### Fostering research integrity: the role of study design

The most critical step in any research is the identification of the study design and the preparation of the written study protocol. The written research protocol is the starting point for good quality research allowing a peer review process by institutional review boards and research ethics committees [70]. According to the document prepared for the International Epidemiology Association Council meeting held in Brazil, April, 2007, [71] "the research protocol is the cornerstone of an epidemiological research project, where the purpose of the study, the hypotheses, the design, the source population, and the planned analyses are described". It is an indisputable fact that it must provide the evidence for the need and feasibility of a research project, a detailed description of the investigation plans, including a clear definition of the study aims and outcomes (e.g., the biological relationships under examination) and the *a priori *hypotheses to be tested within the study along with ethical considerations. These are formulated before the data are collected on the basis of the available scientific evidence as well as the research team experience. A clear differentiation between aims and hypotheses formulated before data collection, and analysis and interpretation of the study findings and their publication thereafter, is central to science. Stating the biological relationships under investigation, the main and secondary research hypotheses, identifying the target population and the study samples, and the statistical methods to be used for formal hypothesis testing is essential. The written research protocol is used by the investigators as a reference tool throughout the study conduct. It prevents potential research misconduct including selective data censoring and torturing (meaning that study data, can be manipulated to prove whatever the investigator likes to prove) [9]. Censoring data to prove or disprove a given hypothesis and torturing data to generate fashionable (statistical) associations of potential biological relevance (deserving further studies and then further funding), can easily be generated from datasets in the absence of a priori declared hypotheses or when a study protocol fails to identify primary and ancillary hypotheses. Negative findings, not supporting the main hypothesis, may result from studies that have not been carefully planned with respect to the number of subjects (i.e., observations) required to efficiently test, statistically, the hypothesis being investigated (i.e., a study with a low statistical power). A study protocol lacking statistical power calculation should not obtain ethical clearance and should not be considered by funding agencies. Moreover, it is important to keep in mind, during the planning phase of a new investigation, that the effect of a poor design cannot be corrected through statistical analysis and that the regular practice of cooperating with statisticians is recommended. Sub-groups analyses (fractionating data), which is usually seen as a way to producing negative results, will almost inevitably result in statistically non significant findings which cannot be interpreted as supportive of the evidence against a study null hypothesis, simply being the result of an inadequate sample size.

## Conclusion

Integrity is central to environmental health research searching for causal relations and requires open communication and trust. Any violation (e.g., data fabrication and falsification, redundant publications, plagiarisms, and undisclosed financial conflict of interest) may endanger the societal trust in the research community. Environmental health research is expected to be scientifically justified, with sound research questions and valid study protocols with sufficient statistical power in epidemiological studies. A clear definition of the study aims and outcomes, of the sample size required to test the  *a priori *identified main hypothesis of this study (according to the defined statistical plan), represents a guarantee for the study conduct and the interpretation of the findings.

Regardless of which human population is included in human studies (e.g., general population, working population, children, elderly, vulnerable sub-groups, etc.) their treatment according to well acknowledged ethical principles must be guaranteed. These principles are aimed at protecting study participants from research-related undesired effects. Study subjects, as well as their biological specimens, and all individual data collected or generated within a study need to be handled in a way that potential physical, mental or social harm can be avoided. Central issues include informing persons being asked to participate in a study, obtaining a written informed consent to participate from those wanting to enrol, and making them aware of their right to refuse or to retract at any time and for any reason. Special ethical aspects arise when children (including newborns) are enrolled in field studies. Given their lack of autonomy up to a certain age there is a need for obtaining their assent together with the permission from their parents or guardians to enrol them in any research. In addition, they should be able to withdraw the consent when they grow older. All these various aspects, but especially the question of privacy, are elemental when establishing and using biobanks, a necessity in environmental health research.

It is the task of us, scientists, to create an atmosphere of trust within the scientific community and more widely in society to be able to establish such biobanks. Strict research integrity, honored by all individual scientists as well as the research community on the whole is the starting point. In addition, justification to public for environmental research is important for people to commit samples and health information, and honors the autonomy of people.

## Competing interests

The authors declare that they have no competing interests.
